# Coupling an agent-based model and an ensemble Kalman filter for real-time crowd modelling

**DOI:** 10.1098/rsos.231553

**Published:** 2024-04-10

**Authors:** Keiran Suchak, Minh Kieu, Yannick Oswald, Jonathan A. Ward, Nick Malleson

**Affiliations:** ^1^ School of Geography, University of Leeds, Leeds, UK; ^2^ Department of Civil and Environmental Engineering, The University of Auckland, Auckland, New Zealand; ^3^ School of Mathematics, University of Leeds, Leeds, UK

**Keywords:** agent-based model, crowd simulation, data assimilation, ensemble Kalman filter, data-driven agent-based modelling

## Abstract

Agent-based modelling has emerged as a powerful tool for modelling systems that are driven by discrete, heterogeneous individuals and has proven particularly popular in the realm of pedestrian simulation. However, real-time agent-based simulations face the challenge that they will diverge from the real system over time. This paper addresses this challenge by integrating the ensemble Kalman filter (EnKF) with an agent-based crowd model to enhance its accuracy in real time. Using the example of Grand Central Station in New York, we demonstrate how our approach can update the state of an agent-based model in real time, aligning it with the evolution of the actual system. The findings reveal that the EnKF can substantially improve the accuracy of agent-based pedestrian simulations by assimilating data as they evolve. This approach not only offers efficiency advantages over existing methods but also presents a more realistic representation of a complex environment than most previous attempts. The potential applications of this method span the management of public spaces under ‘normality’ to exceptional circumstances such as disaster response, marking a significant advancement for real-time agent-based modelling applications.

## 1. Introduction

Agent-based modelling is a technique that was developed as a means of modelling systems that are ultimately driven by discrete, heterogeneous and autonomous agents [1,2] (e.g. people, animals, vehicles, particles, etc.). Rather than attempting to describe systems in terms of their aggregate properties, agent-based models (ABMs) simulate the behaviour of these individual agents directly. This allows modellers to capture many important characteristics that are inherent to the underlying system and cannot be represented with aggregate models [3,4]. Hence, many human systems are well suited to being simulated with ABMs because they avoid the loss of accuracy that can occur when individual-level dynamics are ‘smoothed out’ by aggregate models [5]. Simulations of short-term human movements, such as pedestrian dynamics—the subject of this paper—are no exception. Agent-based modelling has become a particularly popular method for modelling crowds [6] because it captures the individual heterogeneity in a population as well as the interactions between them, both of which are essential for creating reliable micro-level crowd models [7]. Such models may fit within either a theory-based approach, an empirical approach or some combination of the two [8,9]. In a theory-based approach, we may seek to develop theoretical pedestrian models to explore different models of behaviour, while in an empirical approach, we may wish to make use of statistical or machine learning approaches in conjunction with microdata to reflect observed real-world behaviours within our models.

Despite progress in recent decades to improve the robustness of the methodology (e.g. [10]), agent-based modelling still faces a number of challenges (e.g. [3,11,12]) including the understanding and managing of uncertainty [13]—an issue that may particularly impact empirical models that seek to reflect a real-world system. In particular, only a handful of studies have attempted to reduce the uncertainty that will naturally arise as a model and a system evolve [3]. Model calibration is not sufficient for this as even a perfectly calibrated model will diverge from a real system over time. Models are typically calibrated once using historical data and then projected forward in time to make a prediction independently of any new data that might arise. Hence, empirical models that attempt to simulate a system in real time—often referred to as ‘live’ simulations [14]—need a way to update a model state in response to emerging data. Such systems could be transformational in managing a number of different problems. For example, a live simulation of a busy public place such as a public transport hub could allow a manager to better understand how people were moving around and prevent the formation of dangerous crowd bottlenecks. Similar ‘real-time’ systems could revolutionize approaches to disaster response or the spread of disease [14].

This paper makes a contribution that is necessary if ABMs are to become more widely used in policy, particularly for *real-time* applications. Specifically, its main aim is to demonstrate that an ensemble Kalman filter (EnKF)—a method that has shown great value in updating models of physical systems such as the climate [15,16]—can improve the accuracy with which an ABM simulates a system of pedestrians. Although other approaches have attempted to leverage data assimilation (DA) techniques for real-time agent-based modelling [13,17–23], and one has even used an EnKF [24], this paper is the first to apply the EnKF to an ABM that contains unique agents and their interactions (both key elements for an ABM [25]) and tests the algorithm using a real-world example of crowd simulation rather than a toy system.

We present an existing model that can be used to simulate pedestrians in a public transport terminal and show that the model state, that is, the positions of the pedestrian agents, can be updated with new observations as they emerge to keep the model in line with the evolution of the real system.

This paper is structured as follows: §2 reviews the relevant literature; §3 outlines the ABM, the EnKF and the data used; §4 outlines the experiments that will be conducted; §5 outlines the results; and §6 draws conclusions.

## 2. Related works

Most agent-based crowd models (and ABMs more broadly) are essentially models of the past. They are designed to represent a particular scenario and then calibrated and validated using historical data. If the model can successfully reproduce these data, then it is subsequently used to predict the impacts of internal or external changes to the system. Such an approach has theoretical and empirical values—for example, by revealing the underlying mechanisms behind observed crowd dynamics or for general policy or infrastructure planning, respectively—but breaks down if crowds need to be simulated in real time. In this case, various inevitable uncertainties cause even a perfect model to quickly diverge from reality [26]. Despite considerable progress in the area of ABM calibration [10,27], little progress has been made towards methods that can control the uncertainty in models during run time. This makes it hard to predict systems that rapidly evolve, such as pedestrian systems, in real time using ABMs.

An approach that could potentially resolve the issue of real-time model divergence is that of DA. DA has had a variety of uses in the physical sciences; the most notable of these is in weather forecasting, where DA has been one of the key innovations that has caused weather predictions to improve substantially in recent decades [28]. DA refers to a variety of mathematical approaches that allow new observational data to be incorporated into models at run time [29], reducing the uncertainty of the state estimates produced by the models and improving their accuracy. As a consequence of these improved state estimates, models are able to make more accurate future predictions.

A fundamental difference between typical optimization (‘calibration’) and DA is that rather than trying to find optimal model parameter values, DA attempts to estimate the ‘true’ system state, taking into account the model’s current estimate of the state as well as recent observations. It then updates the model state (and, optionally, its parameters) so that the model is closer to the real state of the underlying system. In other words, DA allows the model’s evolution to be constrained against the observations as they arise [24]. Note the difference between the real state of a system and observations of a system—observations are generated through the measurement of the real system state, with the possibility of introducing random and systematic errors; this is a distinction that some previous efforts to implement DA in conjunction with ABMs of pedestrian systems have failed to make [30].

While real-time calibration efforts exist (e.g. [31]), these are not able to reduce the natural uncertainty in the system state that arises as stochastic models evolve. Considering a model of a human crowd, for example, a calibration process may improve the accuracy with which the agents move through an environment. However, natural uncertainty in the behaviour of the people or the occurrence of external events (a person falling over or stopping to look at directions, a train running late, etc.) will cause the simulated crowd to diverge from the real crowd system. This is where DA can fulfil the role of updating a ‘live’ model state in accordance with the real system, specifically the position of individual people in space at any given time.

Although ‘data-driven agent-based modelling’ is not uncommon, relevant approaches usually refer to *dynamic calibration*—that is, re-calibrating a model in response to new data (e.g. [31])—or approaches that adapt agent behaviour in some way—for example, deriving agent behaviour directly from data [32]. Approaches that attempt to update the model state directly, as is the aim with DA, are much rarer.

One of the earliest studies [24] demonstrated that an EnKF could be used to optimize an ABM, although the model had very limited inter-agent interaction so was much less complex than a more traditional ABM. Similarly, attempts have been made to optimize the ‘macro’ model state space, that is, aggregate model characteristics [33], but this ignores local spatial interactions between agents, allowing the model to perform well on an aggregate level but not necessarily at a micro-level. These micro-level interactions are crucial for reliably modelling crowds. Recent attempts have been made to model simple pedestrian behaviour using particle filters [17,20,21] and unscented Kalman filters [23,34]. Although these studies use models that include larger numbers of agents as well as agent–agent interactions, making them more representative of typical ABMs, the environments employed are highly stylized in some cases [21,23,34], and in others, the number and complexity of agent–agent interactions are limited [17]. Entirely novel approaches that adapt theoretical mechanisms from quantum physics [19] or leverage Markov chain Monte Carlo methods [22] are interesting but still in their infancy and not yet appropriate for use in a ‘large’ model; at present, these methods have only been applied to predator–prey models of <100 agents on spatial grids of 32 × 32.

Additionally, there is emerging work in the field of digital twins (DTs) that also makes use of ABMs. The concept of DTs includes a range of simulation approaches that can be run concurrently with the evolution of a physical, social or economic system, aiming to match this evolution [35]. Some such DTs seek to use ABMs to develop digital representations of pedestrian systems [36] that coevolve with the system in real time. As is the case with the ongoing work that seeks to make use of DA, these approaches acknowledge the issues arising from growing levels of uncertainty in models over time and the need for observations of systems to help stave off this growth [37]. Many of these studies, however, neglect the presence of uncertainty associated with observations of the systems in question [38,39]. This is a key consideration, as pedestrian observations may have a degree of uncertainty associated with them [40]. It is, therefore, important that investigations into DTs take this uncertainty into account when developing approaches to interfacing data back and forth between the real-world system and the simulation.

In summary, although progress has been made towards robust DA algorithms, this paper presents a novel approach. It leverages an EnKF—an approach that has been rarely used despite the potential efficiency advantages over the more common particle filter—and also uses a model that has been built to represent a challenging environment, closer to real-world settings, rather than an abstract space.

## 3. Methods

### 3.1. Model and data overview

**Figure 1.**
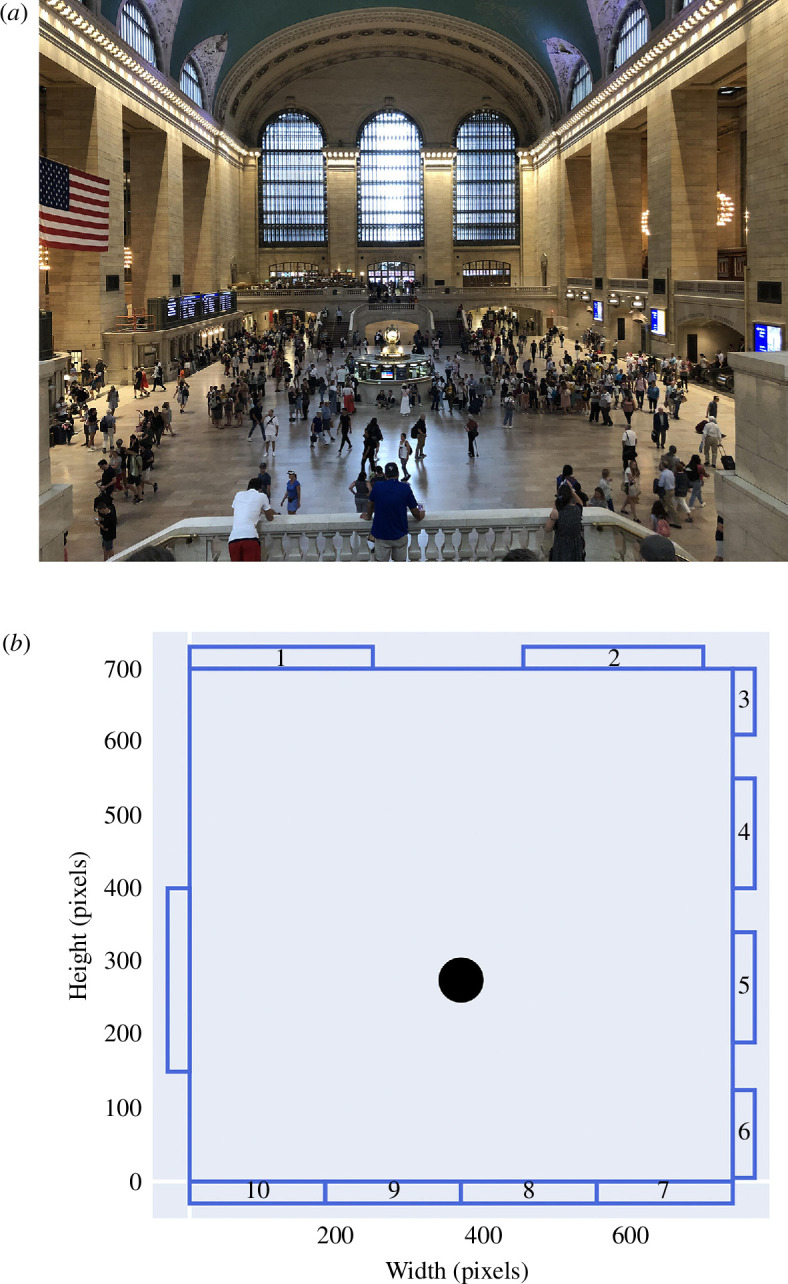
Layout of concourse at GCS in New York. (*a*) Image of GCS concourse from Wikimedia Commons (under a Creative Commons license). (*b*) Diagram of model environment representing the concourse; the black circle represents the information booth.

The model we built is based on the real-world example of Grand Central Station (GCS) in New York, focusing specifically on the concourse area highlighted in figure 1. This model is called ‘StationSim GCS’. It has also been used in work by Ternes *et al*. [21].

The StationSim GCS model is made up of four different types of entities.

- The environment (dimensions specified in table 1).
- Gates around the edge of the environment.
- Obstacles in the environment.
- Agents.

**Table 1.** Table of environment parameters; environment dimensions are provided in pixels, with 1 m being equivalent to 14 pixels.

parameter	value
number of entrances	11
number of exits	11
environment height	700
environment width	740

The environment is portrayed as a two-dimensional continuous space bounded by rectangular walls within which agents may move. Along the boundary are a total of 11 gates that act simultaneously as both entrances and exits (as per figure 1). It also contains an information booth in the centre of the concourse. From a model architecture perspective, this obstacle is treated as a stationary agent; other agents, therefore, treat it as they would treat any other agent and make efforts to avoid colliding with it.

The agents in the model are portrayed as two-dimensional circular entities with a finite radius; each pedestrian agent has the same radius of 7 pixels in model space, equivalent to 0.5 m in real space. During model initialization, each pedestrian in the simulation is assigned an entrance and an exit gate. An agent’s entrance gate is assigned by drawing from a random uniform distribution (all gates have an equal probability of being chosen). Exit gates are assigned in the same way, with the exception that an agent’s exit gate cannot be the same as its entrance gate; an agent’s exit gate is fixed over the course of the simulation. Upon entering the environment, an agent seeks to move as directly as possible towards their assigned exit without colliding with other pedestrians or obstacles. Where collisions are more likely to occur (e.g. close to entrances/exits and around solid obstacles), we typically observe crowding as population densities increase. The variables pertaining to these agents can be found in table 2. The majority of the variables are set to fixed values upon model initialization. Those that change as the model evolves are the location and status variables. The location variable records the current location of the agent; hence, this is updated in every iteration after each agent moves. The status variable is necessary because the number of agents in the model cannot change—a fixed-size state variable is a requirement of our implementation of the EnKF as discussed in §3.3. Hence, all agents that will be needed are initialized before the model starts, but their status is set to ‘inactive’ until they enter the model. Once they enter through their assigned entrance gate they become ‘active’ until they reach the exit gate at which point they become inactive again.

**Table 2.** Table of state variables pertaining to agent entities.

variable name	description
location	agent’s *x*–*y* coordinates in two-dimensional continuous space; bounded by the height and width of the environment
status	agent’s status; 0 indicates agent has not started, 1 indicates agent is active, and 2 indicates agent has finished
size	radius of agent’s circular body
speed	agent’s maximum speed; indicative of the distance covered by an agent in a single time step if unobstructed
unique_id	unique numerical identifier for a specific agent in a model
gate_in	number of the gate through which of the gates the agent enters the environment (0 ≤ gate_in ≤ 10)
gate_out	number of the gate through which of the gates the agent exits the environment (0 ≤ gate_out ≤ 10)
loc_desire	*x–y* coordinate of the agent’s target destination; defined by taking the *x–y* coordinates of gate_out and adding some uniformly distributed random noise

When agents cannot move forward at their set speed because they are obstructed by another agent or obstacle, they exhibit simple ‘sidestepping’ behaviour similar to the tangential evasion heuristic outlined by Seitz *et al*. [41]. This is achieved by randomly choosing to sidestep left or right (i.e. tangential to the direction of travel), with each direction being equally probable. In some cases, this sidestep will not be sufficient to open up a new path for the faster agent as they may be blocked by another slower agent, resulting in them being stuck. While this is a simplification of real obstacle avoidance behaviour, it is sufficient as a means of testing the efficacy of the EnKF algorithm (future work can work towards a more realistic crowd simulation).

**Figure 2.**
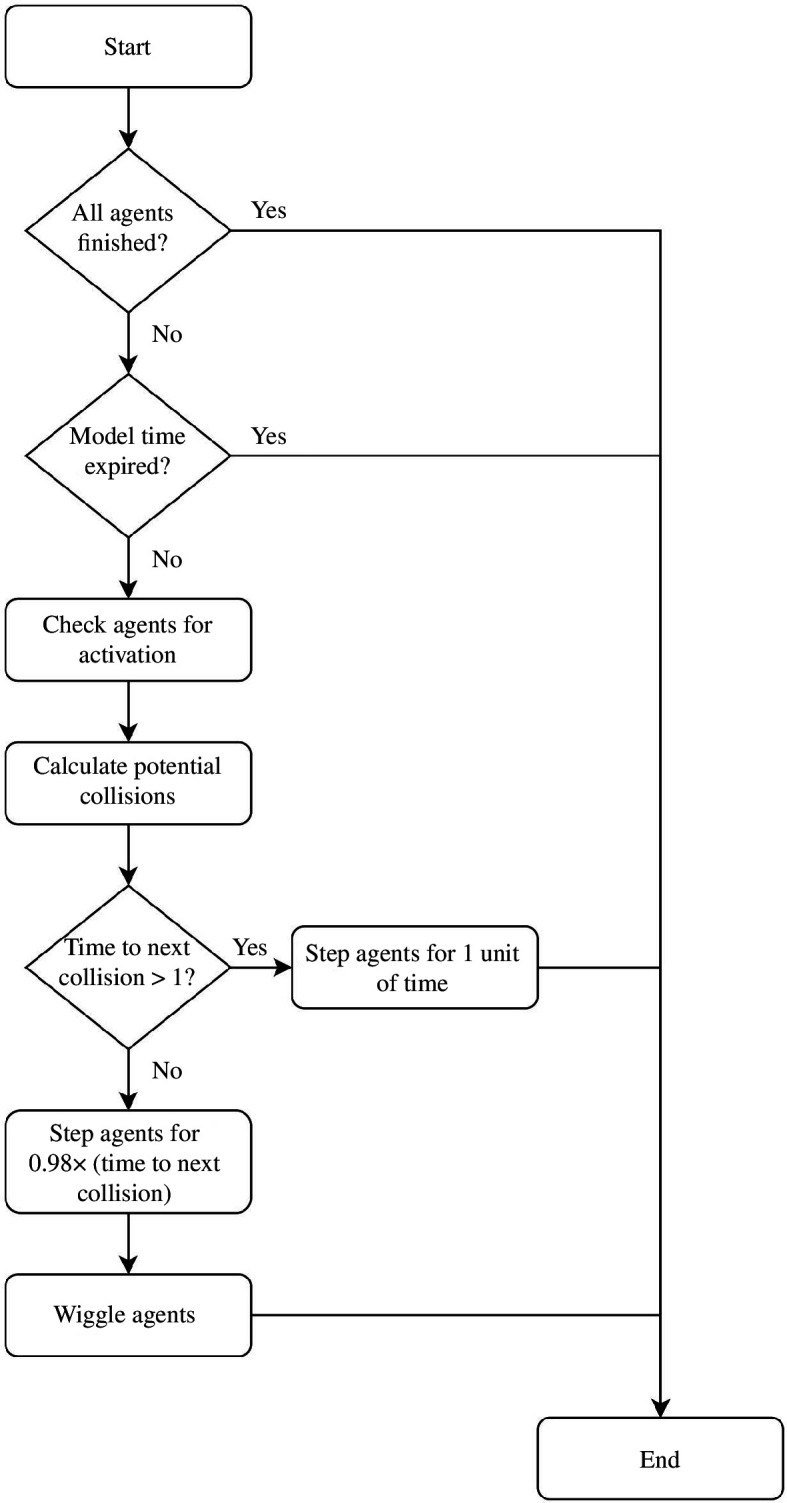
Flow diagram showing the StationSim_GCS model process.

The overall model execution process is outlined in figure 2. The process of initializing the agents in the model involves allocating their entrance and exit gates (as discussed above) as well as their speed and the time at which they will be activated (i.e. when they will attempt to enter the system). The distributions for these parameters are determined in the calibration section below. After having stepped the model forward, it is checked whether there are still some agents who are active in the model; if so, the model stepping process is repeated, else the model run is completed.

The calibration of StationSim_GCS (discussed in §3.2) is undertaken using real-world data captured by cameras at GCS [42]. The raw data published by [42] were cleaned and prepared by [21], resulting in a 2 min video of pedestrian movements. The final data consist of a series of trajectories (a series of *x–y–t* coordinates) for each pedestrian’s location within the environment at the associated timestamp.

### 3.2. Model calibration

Given that the model aims to predict pedestrian movement, calibrating the parameters that are related to movement is central. However, it is important to note that while the model calibration discussed below makes use of real-world data, the later DA experiments make use of pseudo-truth data in order to calculate errors and evaluate model effectiveness, as is common practice for this type of work [17,19,20]. Pseudo-true data are generated by the simulation itself, rather than being drawn from real observations, as will be discussed in §4.

**Figure 3.**
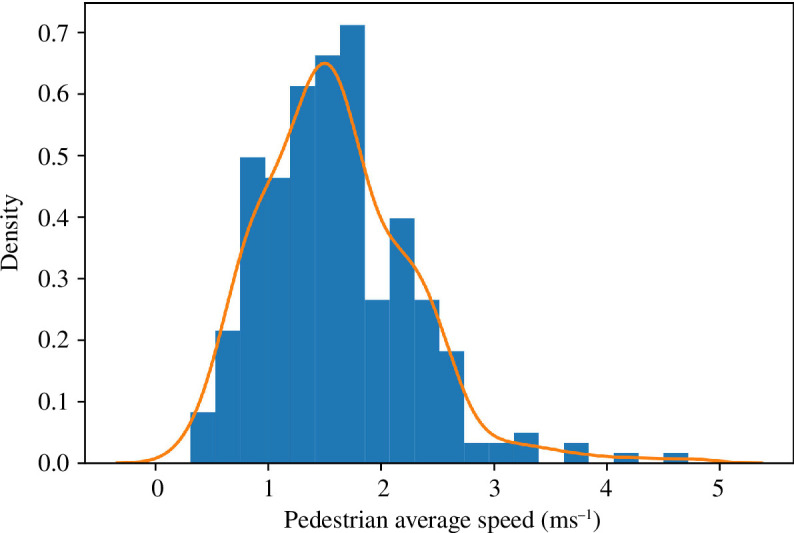
Distribution of average speeds.

A critical parameter is the maximum speed with which pedestrians move (i.e. the speed at which they will move if unencumbered by obstacles). The dataset contains the *x–y* locations for pedestrians in a series of frames. As we know the interval between frames and the true dimensions of the environment, we can calculate the speeds of individual pedestrians. We estimate the distribution of speeds by calculating the Euclidean distance between the start and end points of each pedestrian’s path and dividing by the time it took them to travel across the concourse. While we recognize that this may underestimate the speed as some pedestrians will take convoluted paths to their destination gates, the results are largely in agreement with average pedestrian speeds observed in the literature [43], particularly when considering speeds observed in similar contexts [44]. Figure 3 illustrates the resulting distribution that can be approximated by a mean speed $v_{\mu }=1.60\;{m\;s}^{-1}$ and a standard deviation $v_{\sigma }=0.66\;{m\;s}^{-1}$ . Therefore, in later experiments, the speeds of the agents are drawn from a normal distribution $∼N(v_{\mu },v_{\sigma }^{2})$ . In order to ensure that negative speeds are not drawn from the distribution, a minimum speed is set ( $0.31\;{m\;s}^{-1}$ ), and samples that are below this speed are rejected, prompting a redraw of the speed from the distribution.

The second critical parameter is the rate that agents enter the environment, or the number of agents that are ‘activated’ in a time step. The calibration of this parameter is undertaken via the visual approach of plotting the number of active pedestrians in the system according to the observed data and comparing this to the same data produced by model runs with different birth rates, varying from 1.0 to 2.0 in steps of 0.1 and averaging across 20 model runs for each birth rate. We find that a rate of 1.6 best fits the observed data.

### 3.3. Ensemble Kalman filter

The EnKF is a Monte Carlo variation of the Kalman filter that is particularly useful in situations where the underlying model is complex and nonlinear—as is the case with ABMs. Compared with the usual Kalman filter, the EnKF has several advantages.

**Computational efficiency:** The EnKF uses an ensemble of state estimates that can be propagated independently in parallel, making it computationally efficient for large state spaces.

**Nonlinearity:** It handles nonlinearity more effectively as the state covariance matrix no longer needs to be forecast by applying a linear operator, but instead can simply be generated as a sampling covariance.

The basic idea behind the EnKF is to use a collection (or ‘ensemble’) of possible state estimates rather than just a single estimate. These ensemble members represent different possible evolutionary paths of the system based on different assumptions or uncertainties in the model. At each time step, the EnKF uses the model to predict the state of the system based on the current ensemble of state estimates (termed the ‘predict’ step). It then compares these predictions to actual observations of the system and adjusts the ensemble to better match the observations (the ‘update’ step). The key to the EnKF’s effectiveness is that it uses information from both the model and the observations to update the ensemble. This allows it to handle situations where the model is imperfect or incomplete and where there may be substantial uncertainties in the observations.

The ensemble of system state estimates, X , that consists of a number of individual realizations of the model, x , is defined as


(3.1) $$X=\left[x_{1},...,x_{N}\right]=\left[x_{i}\right],\;\forall i\in \left\{1,2,...,N\right\}.$$

The mean state vector, $\bar{x}$ , can be found by averaging over the ensemble:


(3.2) $$\bar{x}=\frac{1}{N}\sum_{i=1}^{N}x_{i}.$$

As we use the calibrated model to generate multiple sets of pseudo-truth data (discussed in §4), the observations are represented as


(3.3) $$D=\left[d_{1},...,d_{N}\right]=\left[d_{i}\right],\;\forall i\in \left(1,N\right),$$

with each member of the data ensemble matrix, D , being the sum of the original observation d and a random vector, $\epsilon_{i}$ ,


(3.4) $$d_{i}=d+\epsilon_{i},\;\forall i\in (1,N).$$

The random vector is drawn from an unbiased normal distribution:


(3.5) ϵ∼N(0,R),

where R is the data covariance matrix that is defined by the uncertainty in the observations. As with the model state, the mean data vector, $\bar{d}$ , can be found by averaging over the data ensemble:


(3.6) $$\bar{d}=\sum_{i=1}^{N}d_{i}.$$

Given the above framework, the DA is made up of the predict–pdate cycle, with the updating of the state ensemble being undertaken on the basis of the following equation:


(3.7) $$\hat{X}=X+K\;(D-HX),$$

where H is the observation operator. The role of the observation operator is to transform the state vectors between the form in which we store state variables and the form in which they are represented in observations. In the case where we track the *x–y* location of an object in our model and we collect data regarding the *x–y* location of the object, we might have the following scenario:


$$\begin{array}{l} xx={\left[x,y\right]}^{T},\\ d={\left[d_{x},d_{y}\right]}^{T},\\ H=\left(\begin{array}{cc} 1 & 0\\ 0 & 1 \end{array}\right), \end{array}$$

where the observation operator is simply the identity matrix.

In another scenario in which we are modelling the *x–y* location of an object as well as the object’s velocity in the *x–y* plane in our model and we only collect data regarding the *x–y* location of the object, we would instead have the following setup:


$$\begin{array}{c} x={\left[x,y,\dot{x},\dot{y}\right]}^{T},\\ d={\left[d_{x},d_{y}\right]}^{T},\\ H=\left(\begin{array}{cccc} 1 & 0 & 0 & 0\\ 0 & 1 & 0 & 0 \end{array}\right). \end{array}$$

Note here that we do not update our state covariance matrix, instead generating a sample covariance matrix based on the state ensemble, C ,


(3.8) $$C=\frac{1}{N-1}\sum_{i=1}^{N}(x_{i}-\bar{x})(x_{i}-\bar{x}{)}^{T}.$$

The Kalman gain matrix, K , is given by


(3.9) $$K=CH^{T}{\left(HCH^{T}+R\right)}^{-1}$$

in which C is the sample state covariance (used instead of the state covariance matrix Q ) and R is the observation covariance. We can consider (D−HX) in equation (3.7) to be the proposed perturbation to the ensemble states, and the Kalman gain matrix, K , to be the weight given to this perturbation (just as in a standard Kalman filter). When the uncertainty in the observations is low in comparison to the uncertainty in the model state, the gain increases, and therefore the model state receives a larger perturbation from the provided data; conversely, when the uncertainty in the observations is high in comparison to the uncertainty in the model state, the gain decreases, and therefore the model state receives a smaller perturbation from the provided data.

## 4. Experiments

**Figure 4.**
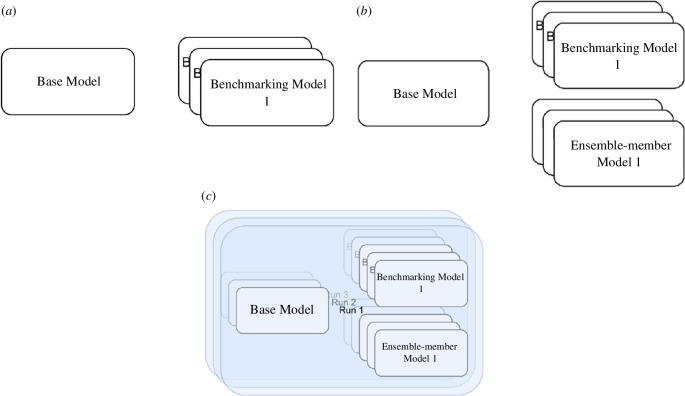
Graphical outline of the three experiments. Note that the ‘base model’ is used to generate pseudo-true observation data. (*a*) Experiment 1: benchmarking, (*b*) Experiment 2: exploring ensemble-member models, and (*c*) Experiment 3: implementing the EnKF.

The experiments, outlined visually in figure 4, aim to demonstrate that the EnKF can improve the accuracy of a pedestrian system in comparison to a baseline scenario with no DA. In order to better understand the impact of DA on an ABM rather than assess the realism of the model itself, we use the ‘identical twin’ approach [18]. In this approach, a ‘Base Model’ is used. A Base Model is an instance of StationSim_GCS that is used to generate ‘pseudo-true’ data that are taken as the real-world observations in the experiments (in lieu of data from a real crowd). When constructing ensembles of models, duplicates of the base model are used in which the corresponding agents are assigned the same entry and exit gates around the environment as well as the time at which they will enter the environment.

The initial experiment (‘benchmarking’, figure 4*a*) seeks to establish a benchmark against which to compare subsequent implementations of the EnKF. This is achieved by running an ensemble of models, each initialized as duplicates of a base model that is used to generate pseudo-truth values for the system state.

The second experiment (figure 4*b*) seeks to demonstrate that the EnKF is effective in reducing both overall simulation error and uncertainty. It does this by exploring the variation in the accuracy of individual ensemble members. This is achieved by running a single EnKF that maintains a benchmarking ensemble of models, providing a baseline against which to compare results, along with an ensemble of models that are periodically updated by the EnKF assimilation process. In such a situation, we are able to compare the average error per agent in each of the ensemble member models at each assimilation time step.

The final experiment (figure 4*c*) takes this exploration a step further by seeking to capture the variation in error at an ensemble level. This seeks to demonstrate that the EnKF is capable of *consistently* improving the accuracy with which we simulate a pedestrian system. This experiment involves running a collection of EnKFs for the same set of model and filter parameters, and in each case, gathering data regarding the variation in the error in the ensemble mean state over time, comparing this with the variation in the corresponding collection of benchmark errors.

### 4.1. Measuring error

#### 4.1.1. Active and inactive agents

As the following sections will discuss, error is calculated by comparing the positions of agents in the simulation with the positions of corresponding agents in the base model (i.e. the pseudo-truth data, discussed in §4). To do this, it is necessary to consider whether an agent is ‘active’ or ‘inactive’ as once an agent has left the simulation, they should not be included in an error calculation. However, an agent might be active in the base (pseudo-truth) model and inactive in some or all of the EnKF ensemble members, or vice versa. Here, we assume that an agent is active only while it is active in the EnKF ensemble because in a real situation, we would not necessarily have access to the true positions of the individuals in the crowd, so could only assess an agent’s status from the information available in the ensemble of models. Hence, an agent is considered active if its most common (i.e. modal) status across the ensemble is active.

#### 4.1.2. Agent-level error

Error at the level of the individual agents is quantified by calculating the distance between the position of an agent estimated by the ensemble of models in the EnKF (the ‘ensemble mean state’) and the position of the corresponding agent in the base model, *d*_*i*_:


(4.1) $$d_{i}=\left\{ \begin{array}{ll} |{\hat{x}}_{i}-x_{i}| & if\;ith\;agent\;is\;active;\\ 0 & otherwise, \end{array}\right.$$

where ${\hat{x}}_{i}$ is the *x*–*y* position of the i th agent estimated by the ensemble of models and $x_{i}$ is the x – y position of the i th agent in the base model. The distance between the ${\hat{x}}_{i}$ and $x_{i}$ agent is calculated using the Euclidean distance.

#### 4.1.3. Model-level error

To calculate the error across the whole system, we calculate the average distance over all active agents in the system, $\bar{d}$ :


(4.2) $$\bar{d}=\frac{1}{N}\sum_{i=1}^{N}d_{i},$$

where N is the number of *active* agents. This average distance, $\bar{d}$ , can then be used to measure the error in an ensemble of models given the ensemble mean state for a given time step and the base model state at the same time step. In this way, we create a base model that is used to generate a ground truth and an ensemble of models from which we can obtain the average behaviour by averaging across the ensemble. The accuracy with which this average behaviour simulates the ground truth generated by the base model is assessed by considering the error between the base model and the average of the ensemble.

### 4.2. Experiment 1: benchmarking

The initial experiment to be performed is to develop a model baseline, establishing the effectiveness of StationSim_GCS in modelling a system in the absence of any information while running. This is applied to ensembles with increasing population sizes (as per table 3) to explore how this behaviour varies with population size.

**Table 3.** Table of model parameters used for estimating the baseline level of error.

parameter	value
population size	[10, 20, 50, 100]
ensemble size	100

In the benchmarking experiments, the following approaches are taken:

- Create an instance of the model to be considered the base model that provides pseudo-truth states of the pedestrian system.
- Create an ensemble of 100 models, each of which is a copy of the base model; this means that each of the duplicates in the ensemble contains the same information regarding which exits each of the agents will enter and exit through, as well as at what time they will be activated within the model. These models, however, are liable to diverge from the base model owing to the collisions that occur between pedestrian agents.
- Iterate each of the base model and the ensemble of models forward for each time step. At each time step, calculate the average model state for each agent in the system population, and calculate the average error per agent between this average model state and the pseudo-truth state generated from the base model for this time step.

### 4.3. Experiment 2: exploring ensemble members

After having established a benchmark for the accuracy with which the StationSim_GCS model can simulate the trajectories of pedestrians moving around the concourse of GCS in New York, the next experiment aims to explore the variation among the models *within* the ensemble of an EnKF while observations are being assimilated into the ensemble. This allows us not only to compare the performance of an instance of the EnKF against the benchmarking ensemble but also to examine the variance within the EnKF’s ensemble of models; the former concerns the accuracy with which we simulate a system while the latter concerns the uncertainty in such a simulation. Simulating a system with an imperfect characterization of the underlying real-world system in the form of a model, stochastic behaviours and inexact estimates of parameter values can all introduce growing uncertainty to model predictions [45]. We, therefore, wish to ensure that uncertainty is minimized. In order to achieve this, rather than calculating the distance between the ensemble mean state and the base model state (as per equation (4.1)), the error is calculated by the distance between each of the ensemble member model states and the base model state. If we consider $d_{ij}$ to represent the distance error of the j th model’s state for the i th agent compared to the i th agent in the base model, this can be calculated as follows:


(4.3) $$d_{ij}=\left\{ \begin{array}{ll} |{\hat{x}}_{ij}-x_{i}| & if\;ith\;agent\;is\;active;\\ 0 & otherwise, \end{array}\right.$$

where ${\hat{x}}_{ij}$ is the x – y position of the i th agent in the j th model and $x_{i}$ is the x – y position of the i th agent in the ground state system, that is, the base model. Given this expression, we adapt equation (4.2) to calculate the mean error per agent for each ensemble member model, ${\bar{d}}_{j}$ , at a given time step as


(4.4) $${\bar{d}}_{j}=\frac{1}{N}\sum_{i=1}^{N}d_{ij}.$$

Based on this, we can construct a vector containing all the mean errors per agent for each of the ensemble member models:


(4.5) $$\bar{d}=\left[{\bar{d}}_{1},\ldots ,{\bar{d}}_{M}\right]$$

(4.6) $$=\left[{\bar{d}}_{j}\right],\forall j\in (1,M),$$

where M is the ensemble size. A vector of average errors per agent for each ensemble member models can then be calculated for each time step.

Based on this error calculation process, we can explore the variation in error across the ensemble using the following steps with the parameter values outlined in table 4:

— Create an EnKF with a population size of 20 pedestrians, containing a base model and an ensemble of 20 duplicates (prior experimentation showed only marginal performance improvements above an ensemble size of 20 members [46]).
— Iterate each of the base models and ensemble of models forward for each time step. If the time step is an assimilation time step, that is, a time step in which synthetic data are produced from the base model, the ensemble of models is updated using the update procedure outlined in §3.3. Assimilation time steps occur with a fixed period of 20 time steps. At each assimilation time step, errors are calculated between the following pairs:
  - The base model state and each of the ensemble member models, as per equation (4.4), after updating with observations.
  - The base model state and the mean state of the ensemble of models, as per equation (4.2), after updating with observations.
  - The base model state and the mean state of the benchmarking ensemble (i.e. the baseline error), as per equation (4.2).

**Table 4.** Table of filter parameters used for the EnKF.

parameter	value
population size	20
ensemble size	20
assimilation period	20
observation noise standard deviation	1.0

### 4.4. Experiment 3: assessing the ensemble Kalman filter

The previous experiments will show that, as expected, the error in the ensemble mean state accurately reflects the variation in error over time and that it does not differ greatly from the error in each of the ensemble member models. On this basis, this final experiment focuses on comparing the variation in the ensemble mean state error over time with the error in the benchmarking ensemble as well as with the error in the observations of the pedestrians’ locations on the station concourse. This considers both the ensemble mean state before and after assimilating observations, that is, the forecast error and the analysis error. The experiment will make use of the parameters outlined in table 5.

**Table 5.** Table of filter parameters used for the EnKF.

parameter	value
population size	20
ensemble size	100
assimilation period	20
observation noise standard deviation	1.0

As with the benchmarking experiment, errors are calculated as the distance between the ‘estimated’ agent location and the agent’s location in the pseudo-truth base model; see equation (4.1). Note that in this case, there are different ways to ‘estimate’ an agent’s location and hence calculate error. We calculate the following errors:

- the base model state compared with the forecast ensemble mean state (i.e. the prior error);
- the base model state compared withthe analysis ensemble mean state (i.e. the posterior error);
- the base model state compared with the observations provided to the ensemble for updating (i.e. the observation error);
- the base model state compared with the mean state of the benchmarking ensemble (i.e. the baseline error).

We use all these methods, and in each case, the distance is calculated in the same manner. Consequently, an average error in each of the four state estimates across all pedestrians can also be calculated in the same manner as per equation (4.2).

We explore the variation in error across the ensemble using the following steps:

- Create an EnKF containing a base model and an ensemble of 20 duplicates of the base model.
- Iterate each of the base model and ensemble of models forward for each time step, updating the ensemble using the DA outlined in §3.3 every 20 time steps, calculating the errors using the four methods above.

This process is repeated 20 times for the same model parameter values and filter parameter values. Each of these 20 cases sets up a different base model and, consequently, a different ensemble of models within the EnKF. The result is that the i th pedestrian agent in the first case may not be identical to the i th pedestrian in each of the other cases and so may be allocated different entrance and exit gates in each case. The aim of this is to ensure that the results produced are not just for a single instance of the EnKF but instead capture the performance of the EnKF in response to different configurations of the pedestrians’ entrance and exit gates (as well as other factors such as speed).

## 5. Results

### 5.1. Experiment 1: benchmarking

**Figure 5.**
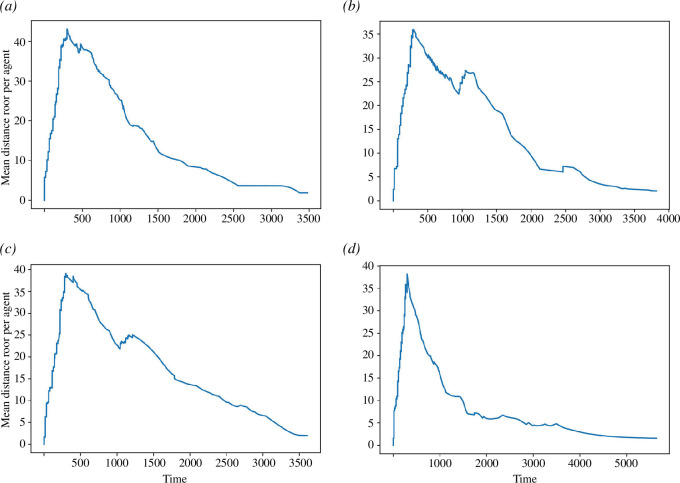
Experiment 1: variation in average error per agent with model time for different population sizes. (*a*) 10 agents, (*b*) 20 agents, (*c*) 50 agents and (*d*) 100 agents.

The results of experiment 1 are shown in figure 5, illustrating how the error varies between a benchmark model (i.e. pseudo-truth data) and an ensemble of 100 models (without DA) as the population size increases. Each of the time-series follows a similar trajectory. The initial error per agent is very low as the starting locations of the agents are known to the ensemble, but it rises sharply as agents begin to move across the environment towards their respective exit gates. The common peak in error is a consequence of the *activation rate* parameter that controls the rate at which agents enter the environment and effectively places an upper limit on the number of agents that can be present in the model at any one time. The peak in error simply at the point in time when the environment contains the largest number of agents.

The error itself is largely attributed to two factors: variations in the precise location along a gate at which an agent enters (although the entrance gate for each agent is known, the exact position within a gate is random) and the number of agent–agent interactions. At lower agent population sizes, the latter is likely to contribute less towards the error with fewer interactions occurring.

In summary, the aim of experiment 1 has been to create a benchmark estimate for the level of error that might be exhibited without DA. Having established this benchmark, the following experiment explores how error varies when DA is implemented.

### 5.2. Results 2: exploring ensemble members

Experiment 2 consists of running a filter that contains an ensemble of models (the ‘filter ensemble’) that undergo DA alongside a benchmarking ensemble of models (the ‘benchmark ensemble’) that have no DA. Both of these ensembles are compared to observations generated by a single base model (the ‘pseudo-truth’ data). This allows us to compare the performance of the filter ensemble against a similar ensemble that has no DA and, importantly, allows us to compare the distribution of errors within the filter ensemble by examining the errors in the individual ensemble models.

#### 5.2.1. Overall filter performance

**Figure 6.**
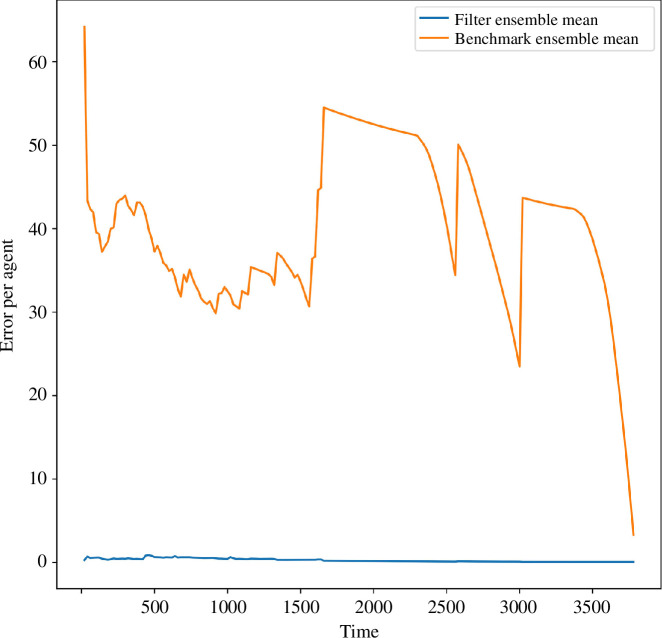
Experiment 2: line plot of the average error per agent based on the mean state of the benchmarking ensemble and the mean state of the filter ensemble.

Figure 6 demonstrates that, as expected, the benchmarking error is much larger than the average error per agent calculated from the filter ensemble mean state. As with previous experiments, the benchmarking error is high at the beginning of the experiment and declines over the course of the filter run; there are, however, some differences between the benchmarking results seen in the two experiments. This is a result of the comparatively smaller ensemble size used for the benchmarking ensemble in this experiment—in this experiment, an ensemble of 20 models was used for the benchmarking ensemble, whereas an ensemble of 100 models was used for the benchmarking ensemble in the previous experiment. Furthermore, as in the previous experiment, we may observe the impact of different frequencies with which data have been sampled; in the previous experiment, data are sampled at every time step and as such we have a relatively smooth line, whereas in this experiment, the data are sampled at every assimilation time step (i.e. every 20 time steps), and so we find noticeable jumps between sequential data points.

When comparing the average error in the benchmarking ensemble against the average error in the filter ensemble mean state, the filter ensemble mean shows a much lower error; consequently, the benchmarking error will be omitted in subsequent figures for this experiment. This demonstrates that the EnKF is effective at using observations to improve the accuracy with which we can simulate a pedestrian system.

#### 5.2.2. Within-filter performance

**Figure 7.**
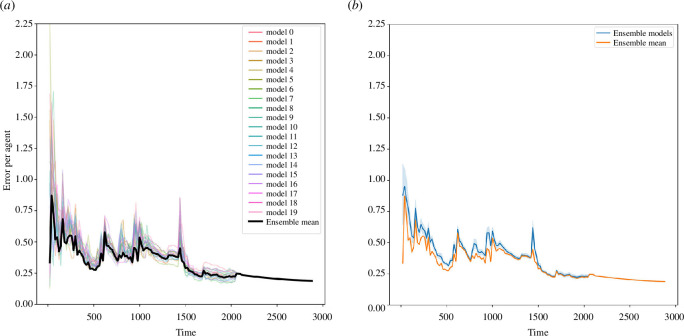
Comparing the ensemble mean error with the errors of the filter ensemble members. (*a*) Error per agent based of each ensemble member. (*b*) Average error per agent from all ensemble member models, with confidence intervals.

We can now explore how the average error per agent varies across the filter ensemble member models and how this compares to the ensemble mean state. This allows us not only to show how the average error per agent varies over time but also to compare the distribution of errors across the ensemble, that is, the in-ensemble variance. This is shown in figure 7*a*,*b*, respectively. In figure 7, the average error per agent is plotted for each ensemble member model as well for the ensemble mean state (plotted in bold black). We can see that the variations in the error in the individual models largely mirror those seen in the ensemble mean state error and that there is relatively low variance across the ensemble. Given that each of the ensemble member models are copies of the base model, this is to be expected. In each of these models, the representation of an individual has the same point of entry and exit around the environment and the same entry time. Consequently, we expect that we will observe the same point in time at which many agents are present in the simulation and are interacting, leading to increases in the simulation error based on the uncertainty driven by these interactions.

**Figure 8.**
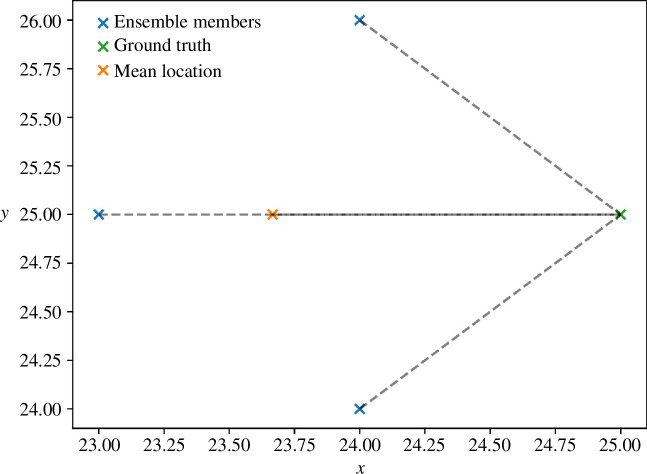
Working example—calculating error based on the ensemble mean compared with the mean error of each individual member models.

It is worth noting that the error in the ensemble mean state appears to typically be lower than the errors in the majority of the individual models. This is further supported by figure 6*b* which, rather than showing the error of the individual models, plots the mean of the model errors and the 95% confidence interval (CI) around it. While we may have expected that these two sets of errors would be identical, this is not the case. However, this is not surprising with hindsight if we consider the hypothetical example illustrated in figure 8. In this example, the error in the ensemble mean is 1.33, while the average of the errors in each of the ensemble member representations is 1.61. It immediately becomes clear that the mean agent location (the ‘ensemble mean’) is likely to have a lower error than the mean of the errors of the individual ensemble members.

In summary, this experiment has demonstrated that the EnKF can not only improve the accuracy with which we simulate a pedestrian system but also with relatively low variance. Furthermore, this experiment has provided an explanation for potential discrepancies between the error in the ensemble mean state and the mean of the error in the individual models.

### 5.3. Results 3: assessing the ensemble Kalman filter

Having established a model baseline level of error and undertaken some exploration of the variation in error across the ensemble-member models within an EnKF, this section completes the experiments by assessing the success of the EnKF as a means of updating an ABM in real time and explores some of the emerging challenges.

#### 5.3.1. Agent behaviour under ensemble Kalman filter

**Figure 9.**
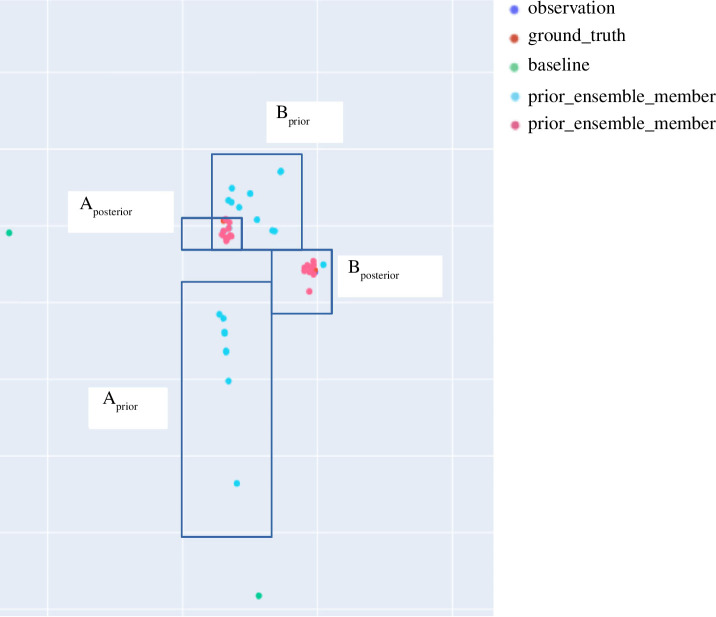
Comparison of prior and posterior positions (in blue and pink, respectively) of two agents (‘A’ and ‘B’) in all ensemble member models.

It is illuminating to explore the behaviour of the individual agents as their state variables are manipulated by the EnKF. To this end, figure 9 illustrates the prior and posterior positions of two individual agents (‘agent A’ and ‘agent B’). We see that the introduction of observations through DA has resulted in the reduction in the spread of the ensemble-member model representations of the agents, that is, the uncertainty in the model estimate of the positions has been reduced. This highlights the effect of the EnKF—not only does it improve the accuracy with which we are able to simulate a pedestrian’s position, but it also reduces the variability in the model’s estimate of the position. This pattern is observed across the other agents in the system.

Having established the ability of the EnKF to reduce the uncertainty in the estimates of pedestrians’ positions in the environment, we tackle a number of additional challenges.

#### 5.3.2. Managing outliers

**Figure 10.**
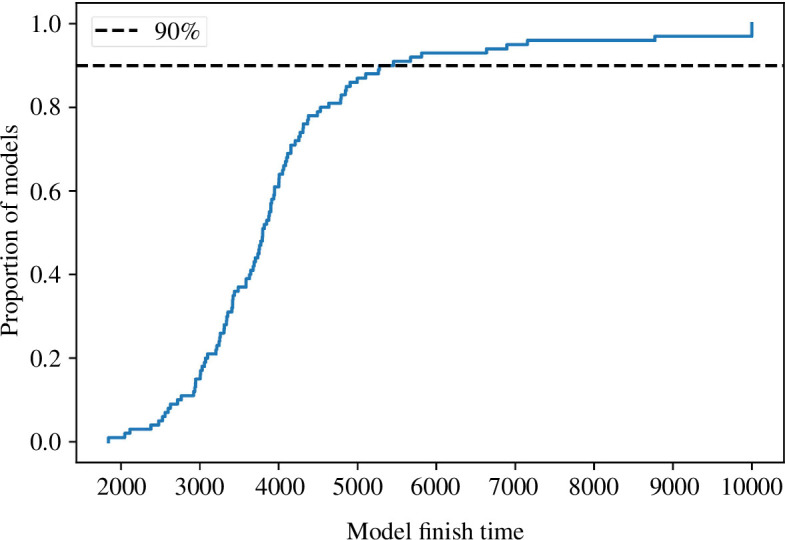
Empirical cumulative distribution function (eCDF) plot of filter finish times; dashed line represents a cumulative level of 90%.

When running a large number of filters with the same filter and model parameters, there is inevitably some variation in the results. This pertains to both the way in which the average error per agent varies over time, and the length of time a filter takes to reach completion. While the instances of the filter may share the same filter and model parameters, this does not mean that they are direct duplicates of each other—the agents within them do not necessarily share the same entry gates, exit gates or entry times. While the majority of the filters reach completion within the first 6000 time steps, which is consistent with the baseline, some do not until approximately 10 000 time steps. This is evidenced in figure 10. Given that the filters are not exact duplicates of each other, we expect that there will be some variation in behaviour of the agents within them resulting from their differing entry and exit points and times. These outliers must be removed as they artificially increase the apparent error of the filters. Hence, when calculating error, time steps after which 90% of the models have completed (typically around 5000–6000 time steps) are disregarded. In addition, the median, rather than the mean, is used to summarize the overall error per agent.

#### 5.3.3. Filter performance

When assessing the average error per agent, three comparisons need to be drawn: (i) benchmarking ensembles against the analysis of the filter ensembles; (ii) the forecast of the filter ensembles against the analysis of the filter ensembles; and (iii) the observations against the analysis of the filter ensembles. This section goes on to explore each of these comparisons. In each case, the comparison is aided by the use of two figures:

- a line plot of the average error per agent over time where the line represents the median of the collection of filters at each time step and the shaded area represents the 95% CI around the line;
- a boxplot showing the distribution of these errors when aggregated over time (logged to reduce the visual impact of the skewed distributions).

**Figure 11.**
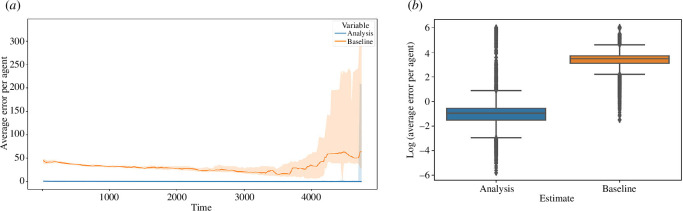
Comparison of average error per agent between analysis and benchmarking filters. (*a*) Line plot of average error per agent over time. (*b*) Box plot of log of average error per agent.

Figure 11 plots the median error per agent by comparing the analysis of the filter ensembles against the benchmarking ensembles. In figure 11*a*, we can see that the average error per agent in the filter analysis states is much lower throughout. We note towards the of the simulation period (i.e. beyond 4000 time steps) that the error in the benchmarking ensembles appears to grow slightly and that the variance across the benchmarking ensembles grows. This is owing to many of the 20 instances having finished their simulation; the instances that complete earlier are typically those that perform better with respect to simulation error, and as such towards the end, we are left with instances that are performing worse, thus skewing the errors and increasing the variance.

This is echoed by the logged boxplot in figure 11*b*. The majority of the logged data pertaining to the analysis error lie below 0, indicating that the average error per agent for the analysis is often below 1. In the case of the benchmarking data, however, the majority of the data lie above 0, indicating that in most cases, the average error per agent for the benchmarking ensembles is much higher.

**Figure 12.**
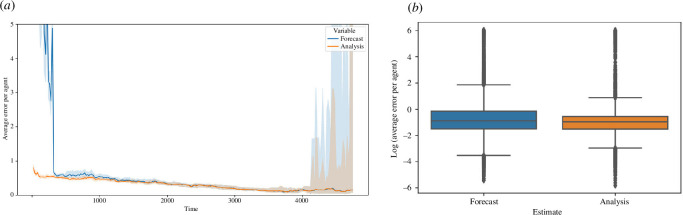
Comparison of average error per agent between analysis and forecast. (*a*) Line plot of average error per agent over time. (*b*) Box plot of log of average error per agent.

Figure 12 compares the average error per agent in the forecast and analysis states of the filter model ensembles, that is, the error before and after assimilating data at each time step. We see that the error in the analysis state is typically an improvement on the error in the forecast state, with the improvements being most noticeable at the beginning of the set of time steps and at the end of the time steps (figure 12*a*). The difference at the beginning is owing to the reasoning outlined in §5.1—the entrance of multiple agents at the beginning of the filter run time and the entry of agents at points on the gates that do not match the exact entry point of corresponding agents in the base model lead to an initial growth in error. The error in the analysis state does not suffer from this growth as it is updated by the provided observations. The increase in the variance of the errors towards the end of the experiment is a consequence of many filters reaching completion and hence the summary statistics being drawn over a decreasing number of filters. Furthermore, figure 12*b* illustrates that, again, the majority of the data lie below 0, indicating that the average error per agent in each case often lies below 1.

**Figure 13.**
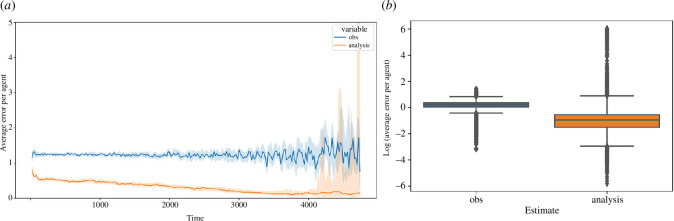
Comparison of average error per agent between analysis and observations. (*a*) Line plot of average error per agent over time. (*b*) Box plot of log of average error per agent.

Finally, figure 13 compares the variation in the average error per agent in the (pseudo-true) observations and the analysis states of the filter model ensembles. The average observation error is largely constant throughout the experiment (figure 13*a*). The increase in error variance is again caused by averaging over a decreasing number of filters. In comparison to the observation error, the analysis error is typically lower for the majority of the time steps for which the filters are running. This is not always the case, however, as highlighted in figure 13*b*. In each case, the errors appear to be low; however, there are substantial outliers pertaining to the analysis error. The observation error, on the other hand, contains relatively few outliers. This is a consequence of how the observations are produced: by adding normally distributed random noise to the base model state.

## 6. Discussion and conclusion

This paper has, for the first time, developed an EnKF that is able to update the individual positions of agents in an agent-based pedestrian model in pseudo-real time. It does this by comparing the pseudo-real positions of the agents (the ‘observations’) to the equivalent positions that are being predicted by an ensemble of ABMs, updating the positions accordingly. The paper demonstrates that the ABM–EnKF system performs considerably better at predicting the positions of the pedestrians than the ABM is capable of doing in isolation. This has important implications for empirical ABMs that are designed to simulate systems in (near) real time, such as those that may form an integral part of a social digital twin [47,48].

Similar attempts to use DA to optimize an ABM in real time have often used variations on the particle filter [13,17,20,21]. However, a drawback with the particle filter is that it typically requires a very large number of particles to ensure that the space of all possible model states is adequately covered, making it extremely computationally expensive. This is demonstrated by table 6, which shows a comparison of the number of ensemble-member models (or particles in particle filter terminology) that have been used in other investigations, and the population sizes that have been simulated. Although a direct comparison with similar work is difficult as the systems and models are different, here an ensemble of only 100 models was adequate to reliably capture the behaviour of the pedestrians in the pseudo-real system. In other work tens of thousands of ensemble members (‘particles’) were required [20]. This is corroborated by work in other fields where DA methods are applied [49]. Given the growing number of investigations in this field, a valuable future investigation may focus on a direct comparison of these methods with a particular focus being given to the importance of factors such as ensemble size, population size and pedestrian behaviours. Such an investigation may also focus on the comparative runtime complexities of the approaches, that is, the way in which the time taken for simulations scales with growth in parameters such as the ensemble size.

**Table 6.** Table of number of particles/ensemble sizes used in similar studies.

study	DA **m**ethod	**p**opulation **s**ize	**e**nsemble **s**ize
Wang & Hu [17]	particle filter	2–6	800–2000
Malleson *et al*. [20]	particle filter	2–40	1–10 000
Ternes *et al*. [21]	particle filter	274	5000
this investigation	EnKF	20	20–100

A drawback with the current approach is that it uses the same ABM to generate ‘real world’ observations (termed *pseudo-true* data) and to predict the behaviour of the system. This means that there is no model discrepancy; that is, no uncertainty between the data-generating mechanism and the simulation. This ‘identical twin’ approach has been used in a number of prior investigations in the field, as well as in other fields in which DA methods are commonly used. In reality, there will always be a degree of model discrepancy and this will undoubtedly influence the accuracy of the predictions of the EnKF. The use of pseudo-truth data is common for exploratory studies [17,20] and has the advantage that we can precisely attribute any uncertainty to its underlying causes, but leaves open the question of how well this approach might work in the real world. Investigations in other fields have compared the use of ‘identical twins’ against the use of ‘non-identical twins’ [50], finding that in those cases the use of ‘identical twins’ resulted in an overestimation of the effectiveness of the DA approach. Immediate future work will begin to test this by incorporating data that are derived from the movements of real people such as Zhou *et al*. [42], along with the use of more complex models of pedestrian behaviour such as a social force model [51]. In addition, the ensemble member models being direct duplicates of the base model (which is used to generate the pseudo-true data) implicitly introduce the assumption that agents’ starting and finishing locations are known to some extent. This is not typically the case. Recent work has demonstrated the impact of removing this assumption [21], and other work has attempted to address this issue by proposing adaptations of traditional DA techniques [23,34]. As yet, however, there is no unified consensus on how to address this issue. Other future work will, therefore, seek to address this within the EnKF approach proposed here.

## Data Availability

The work undertaken in this research paper can be found in 'Projects/ABM DA/experiments/enkf_experiments/results_2/' within this archive of the dust repository [52].
